# Relevance of Rab Proteins for the Life Cycle of Hepatitis C Virus

**DOI:** 10.3389/fcell.2018.00166

**Published:** 2018-12-04

**Authors:** Fabian Elgner, Eberhard Hildt, Daniela Bender

**Affiliations:** Department of Virology, Paul-Ehrlich-Institut, Langen, Germany

**Keywords:** hepatitis C virus, rab GTPases, replication, morphogenesis, release

## Abstract

Although potent direct-acting antiviral drugs for the treatment of chronic hepatitis C virus (HCV) infection are licensed, there are more than 70 million individuals suffering from chronic HCV infection. In light of the limited access to these drugs, high costs, and a lot of undiagnosed cases, it is expected that the number of HCV cases will not decrease worldwide in the next years. Therefore, and due to the paradigmatic character of HCV for deciphering the crosstalk between viral pathogens and the host cell, characterization of HCV life cycle remains a challenge. HCV belongs to the family of *Flaviviridae*. As an enveloped virus HCV life cycle depends in many steps on intracellular trafficking. Rab GTPases, a large family of small GTPases, play a central role in intracellular trafficking processes controlling fusion, uncoating, vesicle budding, motility by recruiting specific effector proteins. This review describes the relevance of various Rab proteins for the different steps of the HCV life cycle.

## Introduction

Hepatitis C virus (HCV) belongs to the genus Hepacivirus of the family *Flaviviridae*. HCV's positive strand RNA genome encompasses 9.6 kb and consists of a 5′-untranslated region (5′-UTR), a long open reading frame encoding a single polyprotein of about 3,010 aa and a 3′-UTR (Simmonds, 2013). Viral and host proteases cleave the polyprotein into the three structural proteins, core that forms the capsid and the two envelope proteins E1 and E2, the viroporin p7 and the non-structural (NS) proteins (NS2, NS3, NS4A, NS4B, NS5A, NS5B). NS3, NS4A, NS4B, NS5A, NS5B form the replicase complexes that are sufficient for the genome replication that does not require the presence of further HCV proteins, enabling the establishment of subgenomic replicons that allow the analysis of HCV genome replication in the absence of infectious virion production (Lohmann et al., 1999). HCV replication occurs in the cytoplasm in the “membranous web” (MW), a characteristic structure for HCV-replicating cells that is formed by reorganization of ER-membranes and lipid droplets (LDs) (Paul et al., 2014; Meyers et al., 2016). While genome replication occurs at the replicon complexes localized at the cytoplasmic face of the membrane network, morphogenesis starts at the LDs that are in close vicinity to the replicon complexes. A characteristic of the mature HCV particle is its heavy association with lipoproteins. In light of this, HCV is frequently described as lipoviroparticle (LVP). In accordance to this, it was assumed that HCV leaves the cell via the lipoprotein pathway, but recent work provided evidence that HCV can be released in form of exosomes via multivesicular body (MVB)-dependent pathways (Elgner et al., 2016; Devhare et al., 2017).

Although there has been a tremendous progress in HCV research the last 20 years, there are still a variety of open questions regarding the molecular virology of HCV. A further more and more relevant aspect is to understand the differences and similarities of HCV to other flaviviruses i.e. Zika virus (ZIKV), Dengue virus (DENV), or West Nile virus (WNV).

Infection by HCV frequently leads to a chronic infection that can cause steatosis, fibrosis, cirrhosis, and hepatocellular carcinoma (HCC). At present there are about 71 million individuals suffering from chronic HCV infection. There is neither a preventive nor a therapeutic vaccine available. The recent development of a new generation of HCV-specific drugs, the direct acting antivirals (DAAs), marks a significant progress to treat and to cure HCV. But the availability of these DAAs is still limited and the response of the different genotypes and the potential development of resistant mutants is not fully clear (da Silva Filipe et al., 2017). Moreover, there is still a variety of open questions with respect to the HCV life cycle.

**Figure 1:**
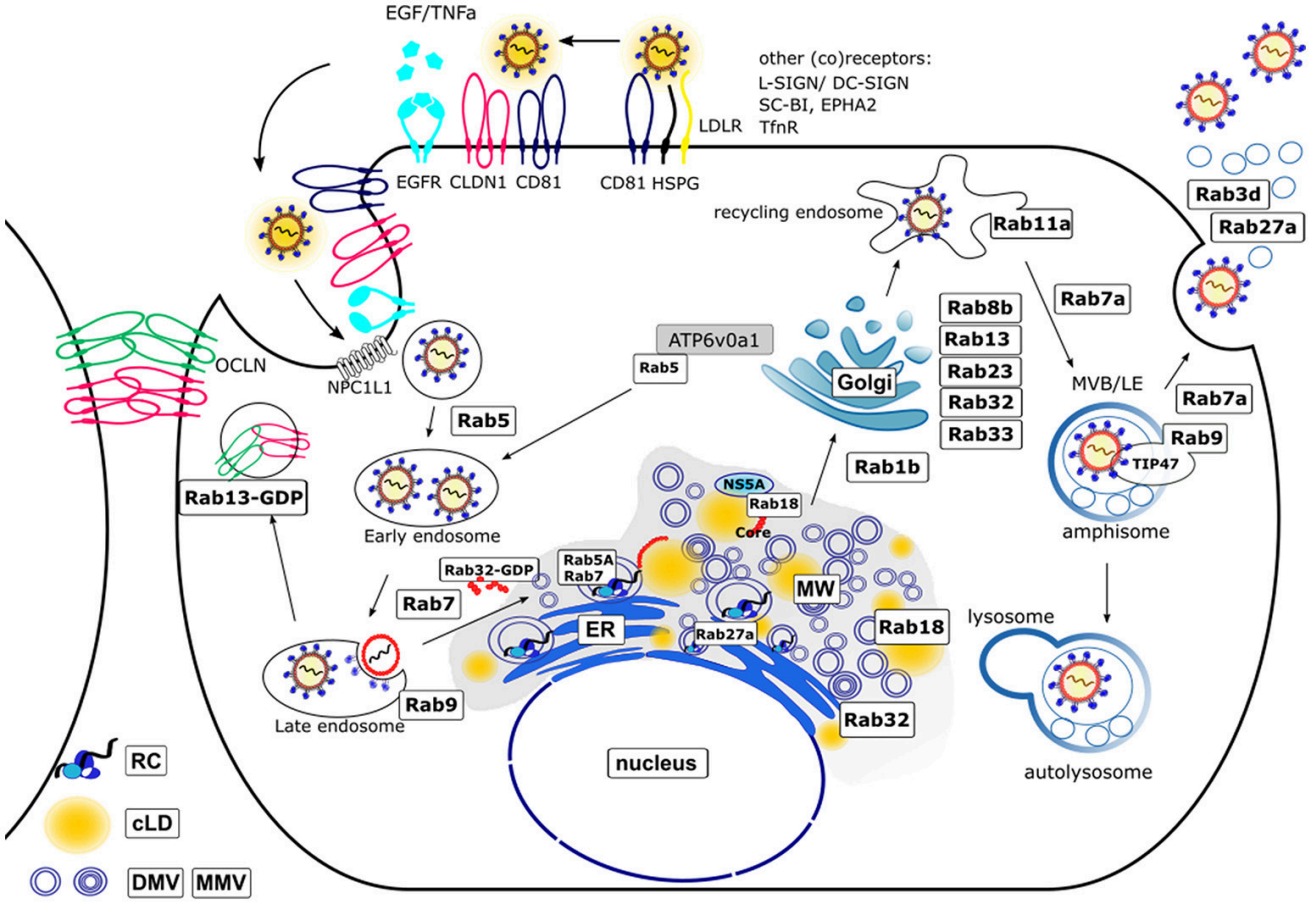
Schematic model of the HCV life cycle and involved Rab proteins. The entry of the HCV lipoviroparticles (LVPs) occurs in a coordinated way by binding of the LVPs to specific (co)receptors. The LVPs initially bind to heparan sulfate proteoglycans (HSPG) and the low density-lipoprotein receptor (LDLR) resulting in a local accumulation. After binding to scavenger receptor class B member 1 (SR-BI) a conformational change of E2 enables the binding to CD81 which then activates signal transduction via the epidermal growth factor receptor (EGFR). The LVP-loaded CD81 migrates by lateral diffusion to the tight junctions (TJ) where interaction with the TJ proteins occluding (OCLN) and claudin-1 (CLDN1) takes place and the LVPs are internalized via clathrin mediated endocytosis [Additional (co)receptors are: L-SIGN, DC-SIGN, ephidrin type A receptor 2 (EPHA2), Niemann-Pick C1-like receptor 1 (NPC1L1) and transferrin receptor (TnfR)]. After maturation of early endosomes to late endosomes the release of the viral nucleocapsid into the cytoplasm occurs in a pH-dependent manner. Hereby, Rab5A is required for acidification as it transports the vacuolar (H+)-ATPase (V-ATPase) ATP6v0a1 from the trans-Golgi (TGN) network toward endocytotic vesicles. The trafficking from early to late endosomes and finally lysosomes for degradation is regulated by Rab5 and Rab7. The TJ proteins OCLN and CLDN1 are recycled under control of Rab13. The uncoated positive-strand RNA is transported to the rough endoplasmic reticulum (ER) where it serves as template for the viral polyprotein synthesis. HCV infection leads to a massive rearrangement of intracellular ER derived membranes leading to the formation of a specific microenvironment, the membranous web (MW). The MW consists of double-membrane vesicles (DMVs) and multi-membrane vesicles (MMVs) embedded in a membranous matrix where HCV replication occurs in replication complexes (RCs). Rab5A and Rab7 are associated with the RCs. Another Rab protein that controls HCV replication is Rab27a. HCV assembly occurs in close proximity to the RCs. Replication and assembly sites are linked via cLDs. The Rab GTPases Rab18 and Rab32 are involved in HCV particle assembly by tethering LDs toward ER derived membranes and facilitating the trafficking of HCV Core and NS5A toward membrane structures involved in particle assembly. The exact mechanism of LVP release remains enigmatic. There is growing evidence that viral particles are released via the endosomal pathway rather than the canonical secretory route. This novel pathway includes the Rab1b-dependent transport of HCV particles through the Golgi compartment. Here, the Rab proteins Rab8b, 13, 23, 32, 33 are involved. Rab11a, a classical marker for recycling endosomes further directs the particles toward late endosomes (Rab7a-dependent) where the particles are sorted either for release by fusion of multivesicular bodies (MVBs) with the plasma membrane (Rab9a and Rab7a-dependent) in parallel to the release of exosomes (Rab3d- and Rab27a-dependent) or for autolysosomal degradation. For this, Rab9 together with TIP47 is required.

Figure 1 summarizes the current knowledge about the involvement of Rab GTPases in the life cycle of HCV.

Rab (Ras-related in brain) proteins are small GTPases of the Ras-like family and regulators of the intracellular vesicle transport. In their inactive GDP (guanosine diphosphate) bound state, they are localized in the cytoplasm and get recruited to cell membranes upon activation due to binding of GTP (guanosine triphosphate). In the active state Rabs interact with distinct effector proteins to fulfill their multiple functions (Eathiraj et al., 2005). Although Rabs possess a weak intrinsic GTP-hydrolysis activity, many Rabs require the enzymatic activity of GAPs (GTPase activating proteins), which hydrolyze GTP to GDP and thereby inactivate the Rabs. GDIs (GDP dissociation inhibitors) are capable to solubilize inactive Rab proteins to provide a pool of activatable Rabs in the cytosol (Pfeffer and Aivazian, 2004). GEFs (guanine-nucleotide-exchange factors) exchange the bound GDP by GTP which leads to an activation of the Rabs. The ability to translocate into membranes is mediated by membrane bound GEFs or geranylgeranylation at the C-terminus of the Rabs. GEFs and GAPs are specific for single Rab proteins or subfamilies and thereby control the molecular switch which regulates fission, transport and fusion of membrane vesicles (Grosshans et al., 2006; Zhen and Stenmark, 2015). Rab proteins regulate amongst others the endosomal pathway, autophagy, and the interconnection of both cellular pathways (Ao et al., 2014).

## HCV Entry

HCV particles are tightly associated with lipoproteins and therefore frequently designated as LVPs (Catanese et al., 2013). The sinusoidal blood stream transports the LVPs through the fenestrated endothelium into the space of Disse (the perisinusoidal space in the liver between a hepatocyte and a sinusoid). Here, an initial interaction with heparan sulfate proteoglycan and LDLR occurs leading to a local accumulation (Jiang et al., 2012; Kobayashi et al., 2012). The activity of lipopropein lipases and hepatic lipases converts very low density lipoprotein (VLDL) to low density lipoprotein (LDL) (Mahley and Ji, 1999). In the next step the LVPs bind to SR-B1 mediated by the high affinity of SR-B1 to E2 and surface exposed cholesterol esters (Zeisel et al., 2007). The SR-B1-bound LVPs accumulate ApoA and ApoC1 from HDL (Acton et al., 1996; Miao et al., 2017). The subsequent conformational change of E2 enables the release from SR-B1 and the binding to CD81, where a conformational change of E1E2 is induced. EGF-R and TGFβ-R are key factors for the control of the subsequent steps (Ströh et al., 2018; Tong et al., 2018). The LVP-loaded CD81 migrates by lateral diffusion to the tight junctions (TJ). Here, the TJs proteins Occludin (OCLN) and Claudin 1 (CLDN1) interact with the complex and thereby mediate a clustering of the tetraspanins (Evans et al., 2007; Ploss et al., 2009). In the next step the complexes that are clustered in the TJs are internalized by inward budding and clathrin-dependent endocytosis (Meertens et al., 2006; Sharma et al., 2011; Miao et al., 2017). The resulting primary endocytotic vesicles undergo a maturation process from early to late endosomes. Hereby, Rab5A is required for acidification (Meertens et al., 2006; Zhang et al., 2011). It is supposed that acidification leads to an irreversible conformational change exposing a so far not definitively identified fusogenic sequence that mediates fusion with the late endosomal membrane and release of the nucleocapsid harboring the viral genome into the cytoplasm (Tong et al., 2017, 2018; Guest and Pierce, 2018; Ströh et al., 2018). Under the reducing conditions of the cytoplasm the capsid disassembles and releases the RNA that enters the replication cycle. There is only limited evidence available describing the function of Rab proteins for the viral entry process.

### Rab5A

The Rab5 and Rab7 GTPases are involved in regulation of cargo trafficking from early and late endosomes to the lysosomes for degradation. The Rab GTPases cycle between a GTP-bound active state and a GDP-bound inactive state. This process is regulated by Rab GEFs and Rab GAPs, respectively. The GTP-bound form of Rab5 and Rab7 is associated with early and late endosomes, respectively, where they are involved in recruiting effector proteins (Wandinger-Ness and Zerial, 2014).

Although the fusion process in the HCV entry process is not fully understood and the fusogenic sequence is still not definitively identified, it is clearly established that HCV entry occurs via receptor mediated endocytosis and depends on endosomal acidification (Tong et al., 2017). The vacuolar (H^+^)-ATPase (V-ATPase) ATP6v0a1 is a central factor in this process (Huotari and Helenius, 2011). The transport of ATP6v0a1 from the trans-Golgi network (TGN) to the endocytotic vesicles is mediated by Rab5A that is enriched in early endosomes. Inhibition of endosomal acidification by Bafilomycin A1 or chloroquine impairs HCV infection in a dose-dependent manner, but has no effect on HCV replication (Blanchard et al., 2006; Matsuda et al., 2014). Real-time imaging experiments using GFP-tagged Rab5A showed CD81- and CLDN1 containing vesicles moving to and co-localizing with Rab5 positive structures. Labeling with EEA1-specific antibodies confirmed the identity of this structures with early endosomes (Farquhar et al., 2012). The detailed role of Rab5A for the virus entry is currently not well-understood.

### Rab13

Rab13 is involved in the regulation of the endocytotic recycling of the TJs-associated proteins CLDN1 and OCLN that are HCV entry factors (Marzesco et al., 2002; Morimoto et al., 2005; Evans et al., 2007; Ploss et al., 2009). Silencing of Rab13 expression impairs the establishment of HCV infection. However, if the expression of Rab13 is silenced after establishment of an HCV infection, there is no impact on the HCV life cycle, indicating that HCV replication is not affected by the silencing of Rab13 expression (Takeda et al., 2016). There is a crosstalk between Rab13, protein kinase A (PKA) and CLDN1 in TJs (Köhler et al., 2004; Farquhar et al., 2008). It was described that PKA promotes the CD81-CLDN1 interaction in TJs. If PKA is blocked, CLDN1 disappears from the TJs and localizes to the cytoplasm. Rab13-GTP binds to PKA and thereby impairs the phosphorylation of CLDN1. The unphosphorylated CLDN1 localizes to the cytoplasm. In contrast to this, Rab13-GDP does not bind to PKA, and PKA phosphorylates CLDN1 enabling the recycling of CLDN1 to the TJs.

The Rab GTPases involved in the entry of HCV virions are summarized in Table 1.

**Table 1:** Rab proteins involved in HCV entry.

**Rab**	**Physiological roles**	**References**	**Role for HCV**	**References**
Rab5a,b,c	Early endosome fusion, early endosome biogenesis, nuclear envelope disassembly in mitosis	Wandinger-Ness and Zerial, 2014	Transport of vacuolar (H+)-ATPase (V-ATPase) ATP6v0a1 from trans Golgi to endocytotic vesicles that is required for acidification	Matsuda et al., 2014 Farquhar et al., 2012
Rab13	Recycling endosome to plasma membrane transport, epithelial junction development, modulation of PKA activity	Marzesco et al., 2002 Morimoto et al., 2005 Köhler et al., 2004	Regulation of the endocytotic recycling of the tight junctions-associated proteins CLDN1 and OCLN	Farquhar et al., 2008 Takeda et al., 2016

## HCV Genome Replication

The disassembly of the nucleocapsid releases the viral plus-strand RNA genome into the cytoplasm. The about 9.6 kb encompassing viral RNA is translated at the rough ER in a single polyprotein with a length of about 3,000 amino acids. It is co- and post-translationally cleaved by viral and cellular proteases in the viral structural and NS proteins (Gu and Rice, 2013; Neufeldt et al., 2018). The core protein and the two envelope glycoproteins E1 and E2 form the viral particle, whereas the p7 viroporin and non-structural protein 2 (NS2) are involved in the virus assembly that occurs on the surface of LDs. The NS proteins NS3, NS4A, NS4B, NS5A, and NS5B, the RNA-dependent RNA polymerase, form the replicase complex. These proteins are sufficient for viral RNA replication and are the base for subgenomic replicon systems (Lohmann et al., 1999). Table 2 lists the Rab proteins with involvement in the viral replication. NS5A, together with host factors, induce massive changes in the ER membrane structure leading to the formation of double membrane vesicles (DMVs). In contrast to previous reports, NS4B seems to be involved in the formation of single membrane vesicles (SMVs). The DMV formation is associated with a massive change in the ER structure leading to the formation of the MW that hosts the viral RNA replicase complexes (Paul et al., 2014; Meyers et al., 2016; Medvedev et al., 2017). The NS5B-dependent genome replication occurs via a negative-strand copy. The *de novo* formed plus-strand RNA copies are either packaged into capsids as viral genome that are subsequently enveloped or are translated in viral polyproteins or serve as template for the synthesis of further minus-strand intermediates (Shi and Suzuki, 2018).

**Table 2:** Rab proteins involved in HCV replication.

**Rab**	**Physiological roles**	**References**	**Role for HCV**	**References**
Rab1	Vesicular targeting and fusion to the Golgi complex by recruiting the tethering factor p115 and v-SNAREs	Allan et al., 2000 Yang et al., 2016	TBC1D20 that acts as the Rab GAP for Rab1 is a binding partner of NS5A, depletion of Rab1 impairs replication by unclear mechanism Post-TGN transport and release of viral particles	Sklan et al., 2007a Sklan et al., 2007b
Rab5a,b,c	Early endosome fusion, early endosome biogenesis, nuclear envelope disassembly in mitosis	Wandinger-Ness and Zerial, 2014	Association with replicon complex; binding to NS4B, relevance for formation of replicon complexes	Matsuda et al., 2014 Farquhar et al., 2012
Rab7a	Endosome maturation; dynein dependent transport of MVBs to lysosomes; fusion of MVBs with lysosomes	Rink et al., 2005 van der Kant et al., 2013 Vanlandingham and Ceresa, 2009	Association with replicon complex;, relevance for formation of replicon complexes	Elgner et al., 2016 Bayer et al., 2016 Wozniak et al., 2016
Rab18	LD-associated; promotes growth of LDs, tethering of LDs to cytoplasmic site of ER Involved in ER-Golgi trafficking ER associated; regulates ER dynamics	Xu et al., 2018 Ozeki et al., 2005 Gerondopoulos et al., 2014	GTP-loaded Rab18 binds to NS5A; Silencing impairs HCV replication	Salloum et al., 2013 Dansako et al., 2014
Rab27a	Secretion of cytotoxic granules; exosome secretion by docking of MVB on plasma membrane	Tsuboi and Fukuda, 2006 Ménasché et al., 2008 Ostrowski et al., 2010	Required for viral replication and post-TGN release of viral particles, effect on miR-122 level	Chen et al., 2015

### Rab5A and Rab7

Rab5A and Rab7 were found to be associated with the replicon complex as characterized by confocal double immunofluorescence microscopy showing a co-staining with NS4B and co-purification with replicon complexes (Manna et al., 2010). Further analyses revealed that silencing of Rab5 and Rab7 expression decreases the *de novo* synthesis of HCV genomes. Although Rab2 was not found to be associated to the replicon complexes, silencing of Rab2 expression decreased HCV replication. In extension of these experiments it was observed that silencing of Rab1, Rab2, or Rab11 had no effect on the formation of replicon complexes. This fits to the observation that coexpression of transdominant negative (tdn) mutants of Rab11 and Rab12 does not affect the formation of replicon complexes (Manna et al., 2010).

This shows the high relevance of Rab5 and Rab7 for the functionality of replicon complexes. However, Rab5 and Rab7 are normally not associated with the ER compartment. On the first glance this seems to be a contradicting observation. In light of this it was speculated that NS4B-bound vesicles are transported to the plasma membrane (PM) and from there retrieved into the cytoplasm via endocytosis leading to the recruitment of Rab5 and Rab7. As inhibition of Rab1 or Rab 2 expression by siRNA or by co-expression of tdn mutants does not affect replicon complex formation and functionality or their association with Rab5 and Rab7, this hypothesis is not likely (Manna et al., 2010). So it is assumed that Rab5/7 are recruited to NS4B to facilitate the fusion of ER-derived vesicles in order to form replicon complexes. This is supported by the observation that Rab5 directly binds to NS4B, but so far there is no evidence for a direct interaction of NS4B with Rab7 (Stone et al., 2007). But in contrast to this model, TBC1D20, that acts as the Rab GAP for Rab1, was identified as a binding partner of NS5A. A region within the amino acids 269–403 mediates the interaction of TBC1D20 with the N-terminal part of NS5A. Based on this it was shown that TBC1D20 depletion inhibits HCV replication and prevents the accumulation of viral RNA (Sklan et al., 2007b). In accordance to this, the same group reported that integrity of Rab1 is required for HCV replication (Sklan et al., 2007a).

HCV replication leads to an induction of autophagy. A central factor for the HCV-dependent induction of autophagy is an elevated ROS level that leads to formation of phosphorylated p62. Autophagy plays a crucial role for the life cycle of HCV. Inhibition of autophagy leads to a decrease in the amount of released viral particles. Expression of tdnRab5 or silencing of Rab5 expression impairs HCV-dependent induction of autophagy and is associated with a decrease in the amount of released viral particles. As described above Rab5 was identified as a binding partner of NS4B. A mutant of NS4B (V233R and L237E) fails to bind Rab5. Rab5 is part of a macromolecular complex encompassing Rab5, Beclin-1, and Vps34 that is involved in the induction of autophagy. In accordance to the observed interaction of Rab5 and NS4B Rab5, Beclin-1, and Vps34 can be coprecipitated with NS4B using a NS4B-specific antibody (Su et al., 2011). Based on this interaction it is assumed that the NS4B/Rab5 interaction is relevant for the HCV-dependent induction of autophagy.

### Rab18

In the HCV life cycle the place of genome replication (replicon complexes) and the place of nucleocapsid assembly (surface of LDs) are separated, raising the question about the efficient, and targeted transfer of the *de novo* synthesized RNA from the replicon complexes to the LDs. NS5A has the capacity to bind the viral RNA (Foster et al., 2010; Hwang et al., 2010). Recent reports described tail interacting protein of 47 kDA (TIP47) as a binding partner of NS5A. Due to the high affinity of TIP47 to LDs and lipoproteins, it is assumed that TIP47 is involved in targeting the RNA-complexed NS5A to the LDs (Ploen et al., 2013a,b; Vogt et al., 2013). Rab 18 was identified as a binding partner of NS5A. The exact binding domain however is not defined. The binding affinity of Rab18 to NS5A depends on the loading with GTP or GDP. The GTP-complexed Rab18 binds with higher affinity to NS5A. Rab18 is found on the surface of LDs and is assumed to promote the association of NS5A with LDs (Salloum et al., 2013). Apart from this there seems to be direct effects of Rab18 on HCV replication and release. The silencing of Rab18 expression decreases HCV replication. The overexpression of Rab18 enhances the release of HCV infectious particles, but does not affect the replication (Salloum et al., 2013; Dansako et al., 2014). From this it can be concluded that the intracellular amount of Rab18 is not limiting for HCV replication. In light of the ability of Rab18 to approximate ER membranes to LDs it is assumed that this contributes to tether NS5A-positive membranes to LDs. In accordance to this it was observed that silencing the Rab18 expression leads to a significant reduction of the proportion of LDs that are poasitive for NS5A and NS3. The relevance of Rab18 for the trafficking of core to the LDs remains unclear (Salloum et al., 2013; Dansako et al., 2014). The capacity of Rab18 to approximate ER membranes to LDs could be relevant for the formation of the MW.

### Rab27a

The HCV genome is stabilized by binding of two molecules of the liver-specific miR-122 that represents a crucial host factor for HCV replication (Jopling et al., 2008; Machlin et al., 2011). Rab27a is known to modulate exosome release by docking MVBs to the PM, an aspect relevant for the release of a variety of viruses including HEV and HCV (Ostrowski et al., 2010; Nagashima et al., 2014; Elgner et al., 2016). But in addition to its effect on HCV release, Rab27a (see chapter release) affects HCV replication (Chen et al., 2015). It was observed that a decreased amount of Rab27a leads to decreased amount of miR-122 most likely by decreasing an abundance of pre-miR-122. The straight forward explanation would be that the loss of miR-122 leads to HCV RNA destabilization, but the replication of HCV genomes harboring stabilizing mutations were affected as well (Sedano and Sarnow, 2014). This indicates that the interplay between Rab27a depletion and decreased amount of intracellular HCV-RNA is more complex and does not exclusively depend on the formation of miR-122/HCV complexes. Although there is no direct experimental evidence, it is speculated that miR-122 has a Rab27a dependent function on the HCV life cycle. It is assumed that this occurs by downregulation of a cellular inhibitor that affects HCV gene expression (Chen et al., 2015).

## HCV Morphogenesis

Infection with the HCV leads to an altered lipid metabolism and rearrangement of intracellular membranes to establish a microenvironment for efficient replication and assembly. The resulting so called “membranous web” (MW) consists of endoplasmic reticulum (ER)-derived DMVs with an average diameter of 150 nm embedded in a membranous matrix (Paul et al., 2014). DMVs are complex structures composed of two tightly associated lipid bilayers highly enriched in cholesterol. However, it is not yet known how the DMVs are formed in HCV infected cells. Due to their similarity to autophagosomes it is assumed that their biogenesis is linked to the autophagosomal pathway. The formation of the MW depends on the interplay of viral and ER-resident proteins. In this regard, the ER-associated GTPases Rab18 and Rab32 are found to be involved in membrane remodeling by tethering LDs toward ER-derived structures. HCV replication mainly occurs in DMVs that contain active viral RNA replicase complexes (Paul et al., 2013), whereas particle assembly takes place in close proximity in detergent-resistant membranes (DRMs) of the ER or mitochondrial-associated ER membranes (MAMs) that are enriched in cholesterol and sphingolipids (Horner et al., 2011, 2015; Shanmugam et al., 2015; Falcón et al., 2017). Cholesterol *de novo* synthesis occurs at the ER, although it shows a low cholesterol carrying capacity. To enrich ER-derived membranes in cholesterol, HCV hijacks the oxysterol binding protein (OSBP)-mediated phosphatidylinositol-4-phosphate (PI4P)-cholesterol exchange or utilizes other lipid transfer proteins, e.g., the Niemann-Pick type C1 protein (de Saint-Jean et al., 2011; Stoeck et al., 2018). Replication and assembly sites are linked via cytosolic lipid droplets (cLDs)—storage organelles for neutral lipids—that act as assembly platform. However, it is not defined whether the assembly proceeds on cLDs itself or on closely associated ER membranes. Particle assembly is a multistep process that requires the crosstalk and concerted action of viral and ER-derived proteins/factors. During the assembly process the newly synthesized viral RNA has to be transferred from the replication complex (RC) to LD-associated HCV Core proteins, followed by nucleocapsid formation and envelopment that occurs by budding into the ER lumen. In conclusion, the formation of the MW enables the virus to establish a spatio-temporal replication and assembly without being recognized by the immune system. In the following we will highlight Rab proteins that participate in HCV particle assembly.

### Rab18

Using Stable Isotope Labeling by Amino acids in Cell culture (SILAC) in combination with mass spectrometry analysis Salloum et al. could identify the LD-associated small Ras-related GTPase Rab18 to interact with NS5A in HCV Jc1-infected Huh7.5.1 cells (Salloum et al., 2013). NS5A is an RNA-binding phosphoprotein that controls the switch between replication and assembly (Zayas et al., 2016; Hsu et al., 2017). It consists of a N-terminal amphipathic α-helix that mediates its membrane association (Brass et al., 2002) and three domains that are separated by low complexity sequences (Tellinghuisen et al., 2004). Domain I and II are mainly involved in RNA replication, whereas domain III participates in virion assembly. Domain III itself contains two regulatory elements: an N-terminal basic cluster that mediates the transfer of the viral RNA to the HCV Core protein and a C-terminal serine cluster essential for transport of the RC to the HCV Core protein (Zayas et al., 2016). Besides, it has been reported that hyperphosphorylation of NS5A domain III by casein kinase 1 (CK1) targets NS5A to cLDs (Tellinghuisen et al., 2008). Rab18 has been identified to be associated with LDs (Martin et al., 2005). In line with this, it is involved in the growth and maturation of LDs through enabling the exchange of lipids between specialized ER regions and tightly associated LDs in so called LD-associated membranes (LAMs) (Ozeki et al., 2005; Xu et al., 2018). Furthermore, it has been linked to ER-Golgi trafficking (Dejgaard et al., 2008). Recently TRAPPII (TRAnsport Protein Particle) has been identified as LD-associated Rab18 GEF. TRAPPII is targeted toward LDs via COPI where it activates Rab18 (Li et al., 2017; Zappa et al., 2017). In addition, the Rab3GAP complex is a Rab18 GEF involved in LD- and ER-targeting of Rab18 thereby controlling ER dynamics (Ozeki et al., 2005; Gerondopoulos et al., 2014; Xu et al., 2018). Mutations in Rab18 and the Rab3GAP complex were found in the autosomal-recessive disorder Warburg Micro syndrome that is characterized by severe developmental abnormalities in the brain and eye, developmental delay, and neurodegeneration (Bem et al., 2011). Moreover, Rab18 impairs the transport of secretory granules in neuroendocrine cells (Vazquez-Martinez et al., 2007) and plays an important role in insulin-mediated lipogenesis and ß-adrenergic-induced lipolysis (Pulido et al., 2011). In hepatocytes Rab18 triggers the association of ApoB100 with LDs after accumulation of free toxic cholesterol (Makino et al., 2016) and links retinoid and cholesterol metabolism thereby mediating hepatic stellate cell activation (O'Mahony et al., 2015). Furthermore, Rab18 enhances HBx-mediated hepatocarcinogenesis through impaired lipogenesis and proliferation (You et al., 2013).

In HCV Jc1-infected Huh7.5.1 cells the direct interaction of NS5A with GTP-Rab18 (activated) promotes the interaction of the MW with LDs thereby connecting sites of replication with sites of assembly. In adipocytes the recruitment of Rab18 toward LDs is mediated via insulin induced activation of PI3K (Pulido et al., 2011). PI3Ks are major effectors of G-protein coupled receptors (GPCRs) or receptor tyrosine kinases (RTKs). Activation of PI3K by growth factors or cytokines generates intracellular phospholipids that in turn activate AKT and other downstream pathways (Liu et al., 2009). HCV NS5A has been reported to bind to and activate the PI3K-AKT pathway. Moreover, Liu et al. reported that HCV transiently activates the PI3K-AKT pathway early after infection to favor viral entry (Street et al., 2005; Liu et al., 2012). Based on these findings, it is tempting to specutlate that HCV might trigger translocation of Rab18 to the surface of LDs through activation of the PI3K-AKT pathway. Using immunoelectron microscopy of HCV replicating cells overexpressing Rab18, the engulfment of thin membrane cisternae around LDs could be detected (Salloum et al., 2013). Rab18 was found to co-localize with NS5A on the surface of LDs. Overexpression of Rab18 resulted in increased amounts of infectious viral particles without affecting viral replication. However, after silencing of Rab18 or expression of a dominant-negative (dn) Rab18 less NS5A as well as the RC components, NS3, and viral RNA could be detected on LDs. In addition, silencing of Rab18 results in decreased amounts of intra- and extracellular viral titers and inhibited viral replication (Salloum et al., 2013). As silencing of Rab18 affects viral replication and assembly it is hard to speculate whether Rab18 indeed affects particle assembly. In line with this, silencing of the LD-associated protein TIP47 similarly inhibits viral replication and release of viral particles. TIP47 binds NS5A and targets the *de novo* synthesized viral RNA to the surface of LDs allowing its interaction with the HCV Core protein, a crucial step during particle assembly (Ploen et al., 2013a). Moreover, interaction of TIP47 with Rab9 is essential for proper release of viral particles (Ploen et al., 2013b) (for more detailed information see chapters replication and release).

For efficient particle assembly the HCV Core protein itself has to be transferred to LDs. During this process the Core protein further recruits NS proteins to the LDs (Miyanari et al., 2007). The HCV Core protein is composed of three domains whereof two proline residues (aa 138, 143) and a YATG-motif in domain II are responsible for its association with LDs (Hope et al., 2002). Association of the HCV Core protein with LDs displaces the LD-associated PAT-protein PLIN2 (or ADRP—adipocyte differentiation-related protein) from LDs and induces their apposition toward the nucleus and ER-derived membranes (Boulant et al., 2008; Counihan et al., 2011). In line with this, overexpression of Rab18 in HepG2 and Balb/C 3T3 cells reduces the amount of PLIN2 on LDs and triggers the encounter of LDs and ER-derived membranes (Ozeki et al., 2005). Silencing of Rab18 in HCV JFH1-infected RSC cells resulted in decreased intra- and extracellular infectious viral particles with no effect on viral replication, which is in contrast to the observations of Salloum et al. Moreover, in immunofluorescence analyses silencing of Rab18 abrogated its co-localization with HCV Core on the LDs and less Core protein was detected around LDs corroborating the role of Rab18 for Core trafficking to LDs. In addition, overexpression of Rab18 increased the production of infectious viral particles two-fold (Dansako et al., 2014). As the observations of both groups differ, the authors explained the contradictory results due to the fact that Salloum et al. used the chimeric HCV Jc1 isolate and different Huh7 subclones. It has been reported that the HCV Jc1 Core protein does not localize to LDs (Shavinskaya et al., 2007) supporting the data of Salloum et al. that could not observe an involvement of Rab18 in HCV Core trafficking.

Taken together, these studies highlight the important role of the small GTPase Rab18 in viral morphogenesis by connecting viral replication and assembly through tethering LDs with ER-associated membranes and trafficking of the HCV Core protein and NS5A from sites of replication toward assembly sites.

### Rab32

Another Rab protein involved in HCV assembly and release is the small GTPase Rab32. In murine melanocytes Rab32 is involved in post-Golgi trafficking of enzymes involved in melanogenesis (Wasmeier et al., 2006). In *Xenopus* melanophores Rab32 has been identified as a melanosome-specific A-kinase anchoring protein (AKAP) that modulates transport of melanosomes by tethering the cAMP dependent PKA toward melanosomes (Park et al., 2007). In non-melanogenic cells Rab32 is localized to ER-membranes and mitochondria and organizes TGN and mitochondria dynamics including mitochondria fission (Alto et al., 2002). Bui et al. reported that ER-resident Rab32 controls Ca^2+^-signaling and modulates MAM-specific characteristics. Moreover, Rab32 tethers PKA to ER and mitochondrial membranes and thereby indirectly regulates the process of programmed cell death via PKA-dependent phosphorylation of enzymes involved in apoptosis (Bui et al., 2010). In addition, ER-associated GTP-Rab32 is involved in phagosome maturation in HeLa and Cos7 cells (Hirota and Tanaka, 2009), intracellular lipid accumulation (Li et al., 2016), and activation of iPSCs (induced pluripotent stem cells) through elevated lipogenesis (Pei et al., 2015), brain inflammation (Liang et al., 2012), and is involved in post-Golgi sorting and transport of LRRK2 (leucine-rich repeat kinase 2) (Waschbüsch et al., 2014) and release of viral particles (Mankouri et al., 2016).

Analysis of the host transcriptome of HCVcc (cell culture grown)-infected Huh7 cells using high-throughput next-generation sequencing (NGS) Pham et al. found 30 genes to be upregulated, including Rab32. In HCV infected cells Rab32 is upregulated on the expression and transcriptional level probably through involvement of HCV NS3 (Pham et al., 2017). Overexpression of Rab32 induces the formation of autophagosomes (Hirota and Tanaka, 2009). As already mentioned above HCV infection results in activation of autophagy via elevated ROS levels and consequent phosphorylation of p62. In line with this, DMVs biogenesis is linked to the autophagosomal pathway corroborating the importance of Rab32 for HCV morphogenesis and the associated rearrangement of ER-derived membranes/MW formation. Interestingly, a shift of GTP-Rab32 to GDP-Rab32 can be observed in HCV-infected cells resulting in intracellular accumulation of GDP-Rab32 making it less susceptible toward degradation. GDP-Rab32 preferentially binds to the HCV Core protein targeting Core toward MAMs (mitochondria-associated membranes) in the perinuclear region where HCV particle assembly takes place. Moreover, silencing of Rab32 resulted in the production of less infectious particles further corroborating its role during assembly and release (see chapter release). Taken together, Rab proteins are involved in membrane dynamics through tethering proteins involved in membrane fusion/fission to their target membranes and finally control intracellular trafficking processes. They are found on various cellular organelles including ER, Golgi, mitochondria, endosomes, and intracellular vesicles (Takai et al., 2001). HCV particle assembly/morphogenesis is still not fully understood. HCV infection is accompanied by a massive rearrangement of intracellular membranes. In line with this, the Rab GTPases Rab18 and Rab32 are found to be involved in HCV particle assembly by tethering LDs toward ER-derived membranes and facilitating the trafficking of HCV Core and NS5A toward membrane structures involved in particle assembly (Table 3).

**Table 3:** Rab proteins involved in HCV morphogenesis.

**Rab**	**Physiological roles**	**References**	**Role for HCV**	**References**
Rab18	LD-associated; promotes growth of LDs, tethering of LDs to cytoplasmic site of ER Involved in ER-Golgi trafficking ER associated; regulates ER dynamics Positive modulator of autophagy and proteostasis Regulates Cholesterol-dependent association of ApoB100 with LDs in hepatocytes Regulates hepatic stellate cell activation Impairs secretory granule transport in neuroendocrine cells Regulates lipolysis and lipogenesis in adipocytes	Xu et al., 2018 Ozeki et al., 2005 Dejgaard et al., 2008 Gerondopoulos et al., 2014 Feldmann et al., 2017 Makino et al., 2016 O'Mahony et al., 2015 Vazquez-Martinez et al., 2007 Pulido et al., 2011	Recruitment of the HCV Core protein to the LDs Binds NS5A; recruitment of LDs to sites of replication	Dansako et al., 2014 Salloum et al., 2013
Rab32	ER- and mitochondria-associated; functions as cAMP-dependent protein kinase (PKA)-anchoring protein (AKAP); controls MAM properties Regulates intracellular lipid accumulation and lipolysis in hepatocytes via adipose triglyceride lipase (ATGL)	Alto et al., 2002 Bui et al., 2010 Li et al., 2016	Interaction of GDP-Rab32 with HCV Core; directing HCV Core toward ER-derived structures in perinuclear region	Pham et al., 2017
	Post-Golgi trafficking of melanosomal enzymes in mice AKAP-dependent melanosome transport in *Xenopus* melanophores	Wasmeier et al., 2006 Park et al., 2007		
	Phagosome maturation in HeLa and COS cells Regulates lipid biosynthesis and activation of autophagy during reprogramming in iPSCs Regulates intracellular lipid accumulation	Hirota and Tanaka, 2009 Pei et al., 2015 Li et al., 2016		
	Involved in brain inflammation Regulates late endosomal transport and sorting of LRRK2 (leucine-rich repeat kinase 2)	Liang et al., 2012 Waschbüsch et al., 2014		

## Release

The egress of infectious HCV particles remains enigmatic. Because of their high similarity to VLDLs regarding morphological and physicochemical properties and the incorporation of lipids and lipoproteins as ApoE and ApoB100, HCV virions are proposed to be LVPs (Catanese et al., 2013). This close functional relationship has led to the assumption that HCV virions may use the canonical secretory pathway to leave the host cells just as VLDLs (Moradpour et al., 2007). However, this hypothesis has never been validated. Quite contrary, recent studies describe differences in the post-ER transport and release between HCV virions and lipoproteins, even though lipoproteins are essential for the proper assembly of the LVPs (Chang et al., 2007; Cun et al., 2010; Bayer et al., 2016; Takacs et al., 2017). Moreover, several key components of the endosomal transport system have been described to play essential roles in the egress of HCV particles, including SNAREs, ESCRT components, and several Rab proteins (Corless et al., 2010; Ariumi et al., 2011; Tamai et al., 2012; Ren et al., 2016, 2017). The later will be discussed here in more detail.

### Rab3d and Rab11a

Using an siRNA-screen Coller et al. identified multiple components of the secretory pathway to be involved in the secretion of HCV virions including proteins required for Golgi trafficking, Golgi sorting, and TGN budding (Coller et al., 2012). Remarkably, knockdown of the exocytotic GTPases Rab3d and Rab11a, which regulate post-TGN to PM transport, strongly decreased the amount of released viral particles. Rab3d is not well-characterized but it has been described to regulate the exocytosis of vesicle cargoes in non-neuronal cells (Millar et al., 2002). It remains unclear, whether this exocytosis represents the release of exosomes by fusion of MVBs with the PM which came into focus just in the past years. Since Rab11a is a classical marker for recycling endosomes (REs), the authors state that trafficking of HCV particles include these sorting organelles (Coller et al., 2012). This is further corroborated by live cell imaging analyses visualizing the HCV Core protein co-trafficking with Rab11a. Knockdown of Rab11a resulted in the accumulation of the Core protein in the Golgi compartment, explaining on the one hand the decreased virion egress and on the other hand validating the involvement of post-Golgi REs in the release route. These results led to the hypothesis, that HCV particles leave the host cell via the secretory pathway, starting at the ER followed by the Golgi, budding from the TGN, and finally the transport via REs to the PM (Coller et al., 2012). Interestingly, the role of Rab11a in the regulation of MVB transport and exosome secretion in human K562 cells and *Drosophila melanogaster* has been described by several groups (Savina et al., 2002, 2005; Fader et al., 2008; Koles et al., 2012). However, whether this function of Rab11a in addition contributes to the proper release of HCV particles has not been discussed by the authors.

### Rab9

With the generation of an E1-mCherry tagged HCV genome, Bayer et al. were able to observe a co-trafficking of the envelope protein E1 with Rab9 (Bayer et al., 2016). Using Fluorescence Recovery After Photobleaching (FRAP) experiments it was confirmed that E1 is present inside membrane enclosed Rab9-positive vesicles, since E1-mCherry fluorescence was not able to recover after bleaching in Rab9-positive loci. The presence of a structural protein does not directly prove the presence of infectious viral particles. However, since there is no regulatory role of E1 known, it can be assumed, that the majority of trafficking E1 resembles assembled HCV virions. A role of Rab9 in the release of infectious particles was further corroborated by recent work from our group. We mutated or deleted the Rab9-binding domains of the intracellular sorting factor TIP47 to inhibit the interaction of these proteins while keeping functional Rab9 available. Besides, TIP47 is a LD-associated protein located on the surface of LDs. In accordance with its role as a sorting factor we could identify that TIP47 recruits the viral RNA bound to NS5A to the surface of LDs contributing to HCV particle assembly, thereby getting incorporated into the viral particle itself. Impairment of the TIP47-Rab9 interaction by expression of the above mentioned mutants did not affect the viral replication but strongly decreased the egress of infectious particles. Moreover, the HCV Core protein accumulates in lysosomal structures (Ploen et al., 2013b). Taken together, these findings highlight a role of Rab9 in concert with TIP47 in the sorting of infectious HCV particles between their release and lysosomal degradation.

### Rab7

Several studies documented the sorting of HCV particles for either lysosomal degradation or release from the host cell (Elgner et al., 2016; Ren et al., 2016). Wozniak et al. found that the amount of the Rab7-dynein adapter Rab-interacting lysosomal protein (RILP) is heavily decreased in HCV infected cells (Wozniak et al., 2016). This comes along with a dramatically shorter half-life of RILP (7 h in infected cells, whereas 62 h in the control cells) and the appearance of a cleavage product of RILP (cRILP) which is missing the dynein-binding motif. The exact mechanism of RILP cleavage remains unclear, but it seems not to be a consequence of apoptosis, ER stress or innate immunity. Interestingly, inhibition of c-Jun N-terminal kinase (JNK) blocks the formation of cRILP also in control cells leading to the hypothesis that RILP cleavage is an intrinsic cellular process. After electroporation of the HCV genome into Huh7.5 cells the virus mainly replicates and assembles, but the release is at a low level for 3 days. At day 5 post electroporation (dpe) the cells convert into an efficient virion secretion state which directly correlates with the appearance of cRILP. The expression of a non-cleavable RILP (ncRILP) 5 dpe reduced the amount of released virions by 60% compared to control cells, whereas cRILP had no effect. In contrast to this, the expression of cRILP strongly increased the amount of released viral particles 3 dpe, but ncRILP had no effect at this time point. This suggests that the cleavage of RILP renders the host cell to be an efficient virion secreting cell. Overexpression of cRILP leads to a delayed vesicular trafficking to lysosomes compared to ncRILP, which is localized perinuclear in vesicular structures. Inhibition of kinesins abolished the cRILP effect and rescued intracellular virion levels back to control values. This indicates that cRILP renders Rab7-containing vesicles to bind to kinesins which traffic anterograde to the cell periphery to execute virion release. In contrast, full length RILP promotes binding of Rab7-containing vesicles to dyneins moving retrograde to fuse with lysosomes ending in the degradation of the cargo (Wozniak et al., 2016). This study is an example of how viruses exploit also Rab effector proteins to modify Rab protein functions for their benefit.

Rab7 is involved in the maturation of early to late endosomes and regulates the fusion of the later with autophagosomes and lysosomes (Rink et al., 2005; Vanlandingham and Ceresa, 2009). Rab7-positive vesicles were also observed to co-traffick with the above mentioned E1-mCherry HCV virus in live-cell imaging studies corroborating the role of Rab7 in the release of infectious particles (Bayer et al., 2016). In addition, our group observed in a recent study that treatment of HCV positive cells with the substance U18666A leads to the formation of large multilaminar bodies (MLBs) positive for Rab7, the lysosomal protein LAMP2 and the tetraspanin CD63 which is a common exosome marker (Elgner et al., 2016). U18666A is described as MVB/cholesterol-transport inhibitor and fulfills this function by direct inhibition of the cholesterol transporter NPC1 which is resident in the MVB membrane (Appelqvist et al., 2011). Inhibition of NPC1 by U18666A results in an accumulation of cholesterol in these organelles, sensed by ORP1 resulting in a dynein-dependent transport of MVBs to lysosomes (Rocha et al., 2009). The fusion of lysosomes with cholesterol overloaded MVBs triggers the formation of lysosomal storage compartments, MLBs, which are not degradation competent. Interestingly, MLBs were also found to include E1, ApoE, and neutral lipids highlighting the role of Rab7-positive MVBs in the egress of HCV VLPs (Elgner et al., 2016).

### Rab27a

Regarding the impact of Rab7a and Rab11a, there is raising evidence, that HCV particles might use an exosome-like release pathway. Indeed, knockdown of the exosome controlling Rab27a by Shrivastava et al. reduced the amount of released exosomes and HCV virions. As knockdown of Rab27a also diminished HCV replication, no direct influence of Rab27a knockdown on the release of HCV particles should be stated here (Shrivastava et al., 2016).

### Rab1b

In a lentiviral large-scale screening including 62 dominant negative (dn) Rab mutants Takac et al. observed that dn versions of Rab35, Rab8a, Rab13, and Rab43 reduced the secretion of ApoE, ApoB100 and albumin whilst dn mutants Rab8b, Rab11a, Rab11b, and Rab12 augment the secretion of these proteins (Takacs et al., 2017). Most interestingly dnRab1b reduced the secretion of ApoB100 and stimulated that of ApoE whilst dnRab23 inhibited secretion of albumin and ApoB100 and stimulated ApoE release. These differential results suggest that the investigated cargoes use distinct routes to leave the cell. The authors focused on the characterization of Rab1b and generated two mutants of the protein. Rab1b_S22N_ is locked in its inactive GDP-bound form and Rab_N121I_ is unable to bind nucleotides. Therefore, both mutants are potent ER-to-Golgi transport inhibitors. Rab_N121I_ impaired the secretion of all three tested cargoes, confirming that their secretion involves Rab1b function. The mutant Rab1_S22N_ inhibits the secretion of albumin and ApoB100, but not that of ApoE and the same phenotype is seen by Rab1b inhibition by the bacterial protein DrrA. This difference can be explained by the fact that Rab1b functions at several stages of ER-to-Golgi transport: ApoB100 and albumin may exit the ER in a Rab1b-dependent process whereas ApoE does not require Rab1b to leave the ER (Plutner, 1991; Schwaninger et al., 1991). In a later step, during ER-to-Golgi transport, all three cargos may depend on Rab1b function to reach the Golgi. To investigate the role of the Rab1b mutants in the release of HCV particles, a TetON system was used, avoiding proposed Rab1 effects on the viral replication. The expression of the Rab1b inhibitors was induced 2 dpe, when the viral replication machinery has been established. The expression of DrrA, Rab1b_S22N_, and Rab1b_N122I_ resulted in ER-retention and thus an impaired release of infectious viral particles. This reflects the effect of the Rab1b inhibitors on the secretion of ApoB100 but not that on ApoE, depicting that HCV particles rely on the early function of Rab1b facilitating ER exit. Therefore, despite the correct assembly and infectivity of HCV virions depends on ApoE, they use distinct routes that are differentially regulated release pathways (Takacs et al., 2017).

### Exocytotic Rabs

Another screen with dn mutants of several Rab proteins identified the secretory/exocytotic Rab proteins Rab8b, 13, 23, 27, 32, and 33 to be required for HCV particle release, whereas the endocytotic/recycling GTPases Rab7, 9, 11, and 35 did not seem to play a role in virion egress (Mankouri et al., 2016). This is in contrast to previous studies, as discussed above. The authors explain the different observations due to the fact that they use the original JFH-1 isolate which poorly replicates and releases infectious HCV particles compared to the chimeric virus Jc1 used in the other studies. However, according to the post-TGN-transport functions of the above mentioned Rab proteins (Rab8b, 13, 23, 27, 32, and 33), the authors claimed that assembled HCV particles transit the Golgi compartment on the way to the defined secretory endosomal pathway. Remarkably, none of the dnRab proteins impaired the release of ApoB and ApoE further contributing to the theory that HCV virions and lipoproteins leave the cell by distinct routes, suggesting a post-release association of VLDLs and HCV particles, rather than the formation and secretion of a hybrid LVP.

Taken together, since several years there is growing evidence that HCV is using an endosomal pathway for the release of infectious particles rather than the canonical secretion pathway. In addition, this HCV-release pathway might share some commonalities with the secretion pathway of VLDLs, including the lipoproteins ApoE and ApoB. HCV exploits several Rab proteins and Rab effector proteins to establish or modulate this pathway for its own benefit as summarized in Table 4. Currently based on the above described data, a novel model pathway could be established including the transport of HCV virions from the ER to the Golgi compartment (Rab1b) followed by transport of the virions through the Golgi compartment and TGN (Rab8b, 13, 23, 32, 33) into REs (Rab11a) and finally to late endosomes (Rab7a). In this sorting platform the virions may be sorted for either lysosomal degradation or release by fusion of the MVB membrane with the PM (RILP, Rab7a, Rab9a) which occurs in parallel to the release of exosomes (Rab3d, Rab27a). The Golgi compartment may represent a crossing point for the HCV-release pathway and the canonical pathway where the HCV virions and the VLDLs might get in contact to form the proposed LVPs. In addition, an HCV egress pathway completely independent from the Golgi compartment has been proposed (Bayer et al., 2016). Whether this Golgi-bypass is an additional- or the main viral release pathway needs further investigation.

**Table 4:** Rab proteins involved in HCV release.

**Rab**	**Physiological roles**	**References**	**Role for HCV**	**References**
Rab3d	Regulates exocytosis is non-neuronal cells	Millar et al., 2002	Exocytosis of viral particles	Coller et al., 2012
Rab5a,b,c	Early endosome fusion, early endosome biogenesis, nuclear envelope disassembly in mitosis	Wandinger-Ness and Zerial, 2014	Transport of vacuolar (H+)-ATPase (V-ATPase) ATP6v0a1 from trans Golgi to endocytotic vesicles that is required for acidification	Matsuda et al., 2014 Farquhar et al., 2012
Rab7a	Endosome maturation; dynein dependent transport of MVBs to lysosomes; fusion of MVBs with lysosomes	Rink et al., 2005 van der Kant et al., 2013 Vanlandingham and Ceresa, 2009	Virions are transported in MVBs; cleaved RILP enhances kinesin-dependent release of virions from Rab7 containing vesicles	Elgner et al., 2016 Bayer et al., 2016 Wozniak et al., 2016
Rab8b	Apical post-TGN transport; basolateral TGN to recycling endosome transport	Sato et al., 2014 Henry and Sheff, 2008	Post-TGN transport and release of viral particles	Mankouri et al., 2016
Rab9a	Transport of Mannose 6-phosphate receptor from endosomes to Golgi	Lombardi et al., 1993	Required for TIP47-dependent release rather than lysosomal degradation of virions; virions cotraffick in Rab9a-positive compartments	Ploen et al., 2013b Bayer et al., 2016
Rab11a	Transport through recycling endosomes; transport from TGN to plasma membrane; homotypic MVB fusion; exosome release; amphisome formation	Ullrich et al., 1996 Chen et al., 1998 Savina et al., 2005 Savina et al., 2002 Koles et al., 2012 Fader et al., 2008	Post-Golgi sorting of virions in recycling endosomes	Coller et al., 2012
Rab13	Regulates tight junction structure and function; TGN to recycling endosome transport Recycling endosome to plasma membrane transport, epithelial junction development, modulation of PKA activity	Marzesco et al., 2002 Nokes et al., 2008 Morimoto et al., 2005 Köhler et al., 2004	Post-TGN transport and release of viral particles Regulation of the endocytotic recycling of the tight junctions-associated proteins CLDN1 and OCLN	Mankouri et al., 2016 Farquhar et al., 2008 Takeda et al., 2016
Rab23	Regulator of sonic hedgehog signaling; autophagosome formation	Evans et al., 2003 Nozawa et al., 2012	Post-TGN transport and release of viral particles	Mankouri et al., 2016
Rab27a	Secretion of cytotoxic granules; exosome secretion by docking of MVB on plasma membrane	Tsuboi and Fukuda, 2006 Ménasché et al., 2008 Ostrowski et al., 2010	Required for viral replication and Post-TGN release of viral particles	Shrivastava et al., 2016 Mankouri et al., 2016
Rab32	Post-Golgi trafficking of melanogenic enzymes; mitochondrial fission; phagosome maturation	Wasmeier et al., 2006 Alto et al., 2002 Seto et al., 2011	Post-TGN transport and release of viral particles	Mankouri et al., 2016
Rab33	Retrograde intra-Golgi transport and from Golgi to ER; modulates autophagosome formation	Valsdottir et al., 2001 Starr et al., 2010 Itoh et al., 2008	Post-TGN transport and release of viral particles	Mankouri et al., 2016

## Conclusions and Future Perspectives

Although there has been a tremendous increase in our knowledge about HCV, mainly based on the establishment of subgenomic (Lohmann et al., 1999) and full length genomic (Blight et al., 2002; Wakita et al., 2005; Zhong et al., 2005) replication systems, there are still a variety of open questions regarding the HCV life cycle. This concerns the understanding of the entry process and characterization of the fusion process or the release of HCV with respect to the relevance of MVB-dependent processes and the release in form of exosomal vesicles. It should be considered that the biggest part of our knowledge is based on the use of immortalized hepatoma cell lines and a very limited number of isolates. In light of this, the comparative analysis of various genotypes with respect to their dependency on Rab-mediated processes and the use of primary hepatocytes will be challenging. Our present view on the molecular virology of HCV is in many aspects a static view i.e., based on the analysis of fixed cells. Just with respect to the central role of RabGTPases in controlling trafficking processes, live cell imaging to investigate the spatiotemporal regulation of the HCV life cycle will contribute to a deeper understanding of the pathogen host interaction.

In light of the central role of RabGTPases for a variety of cellular processes targeting of Rab GTPase to control HCV replication seems not to be a very promising approach since a variety of side effects can be expected. Strategies directly affecting the interaction between viral proteins and Rab GTPases e.g., by the development of small molecules or competitive peptides that are pathogen specific will be more straightforward. However, different strategies to target GTPase activity have been under investigation. One approach is to address post-translational modifications by inhibition of lipid transferases (farnesyl- and geranylgeranyltransferases) that tether GTPases toward their target membranes. Albeit, clinical trials reported about toxicities of these compounds making them less attractive for therapeutical applications (Konstantinopoulos et al., 2007). Moreover, inhibition of the mevalonate pathway interferes with the biosynthesis of isoprenoids [farnesylpyrophosphate (FPP) and geranylgeranylpyrophate (GGPP)], which are substrates for prenylation, and cholesterol synthesis. In context of HCV, statins, inhibitors of the 3-hydroxy-3-methylglutaryl coenzyme A (HMG-CoA) reductase in the mevalonate pathway, inhibit HCV replication, and were reported with a lower risk of HCC (El-Serag et al., 2009) and lower portal pressure in patients with cirrhosis (Abraldes et al., 2009). Treatment with statins inhibits the geranylgeranylation of host proteins that play a crucial role during the HCV life-cycle via inhibition of GGPP and not cholesterol (Ikeda et al., 2006, 2011). Another possibility to modulate GTPase activity is to regulate their function by addressing GAPs, GEFs, and GDIs. Recently, the small molecule pan-GTPase inhibitor CID1067700 has been identified to inhibit multiple GTPases (Agola et al., 2012; Hong et al., 2015). However, the specific application of inhibitors and their progress in clinical trials is limited. In addition, the small globular structure of GTPases and their high affinity toward ligands depicts GTPases as difficult target for inhibtors (for more detailed information see Konstantinopoulos et al., 2007).

HCV belongs to the family of *flaviviridae* that encompasses a variety of emerging viruses like ZIKV or WNV. Thus it will be interesting to see whether insights obtained for HCV can be transferred to other flaviviruses and allow the development of antivirals with a “pan-flavivirus” spectrum.

## Author Contributions

All authors listed have made a substantial, direct and intellectual contribution to the work, and approved it for publication. The authors contributed equally to all sections of this work. EH focused on HCV entry and replication, DB on morphogenesis, and FE on release. DB illustrated the figure.

### Conflict of Interest Statement

The authors declare that the research was conducted in the absence of any commercial or financial relationships that could be construed as a potential conflict of interest.
